# Outcomes from a new modified single needle laparoscopic percutaneous extraperitoneal closure and cut off for pediatric inguinal hernia

**DOI:** 10.1038/s41598-024-62769-7

**Published:** 2024-05-26

**Authors:** Defeng Zeng, Changsheng Pu, Chunbao Guo, Xiangpan Kong

**Affiliations:** 1 Department of pediatrics; Women and Children’s Hospital of Chongqing Medical University, Chongqing, People’s Republic of China; 2 Department of Pediatric Surgery, Chongqing Health Center for Women and Children, Chongqing Medical University, Chongqing, 400 054 People’s Republic of China; 3 Department of Urology; Ministry of Education Key Laboratory of Child Development and Disorders; National Clinical Research Center for Child Health and Disorders; China International Science and Technology Cooperation Base of Child Development and Critical Disorders, Children’s Hospital of Chongqing Medical University, Chongqing, People’s Republic of China

**Keywords:** Inguinal hernia, Pediatric, Patent processus vaginalis, Laparoscopic hernia repair, Minimally invasive surgery, Paediatrics, Therapeutics

## Abstract

Inguinal hernia is a prevalent surgical condition in pediatric patients. Despite the efficacy of current treatment modalities, a certain recurrence rate still persists. Hence, our objective in this study is to introduce an innovative surgical technique designed to minimize surgical complications. We conducted a retrospective analysis on 809 pediatric cases that underwent laparoscopic repair with our innovative technique for inguinal hernia from June 2020 to June 2022. Demographic information, perioperative details, and postoperative follow-up outcomes were thoroughly assessed. All surgeries were conducted laparoscopically under general anesthesia. The procedure commenced by encircling the hernia sac with two sutures under laparoscopic guidance. Subsequently, the sac was exteriorized from the body using the two sutures, followed by ligation and excision of the hernia sac. The research findings demonstrate that the duration of unilateral and bilateral procedures was recorded as 15.9 ± 4.8 and 21.7 ± 3.9 min, respectively. Incision infection occurred in 7 patients (0.87%), and Male Complicated Inguinal Hernia (MCIH) was observed in 2 patients (0.23%). Notably, there were no occurrences of iatrogenic cryptorchidism, testicular atrophy, or recurrence (0%) during the follow-up period. In conclusion, our novel modification shows a notable reduction in postoperative recurrence rates and alleviates the impact of the procedure on the positioning of the testis or uterus. This modified technique is both safe and valuable, thus warranting broader adoption and promotion.

## Introduction

Inguinal hernia is one of the prevalent conditions in pediatric surgery, exhibiting an incidence ranging from 0.8 to 4.4%^1^. Consequently, the surgical repair of pediatric inguinal hernia has become a commonplace procedure in the field of pediatric surgery. With over two decades of development, laparoscopic inguinal hernia repair (LIHR) has garnered increasing acceptance globally. Recognized for its advantages such as clear anatomical delineation, favorable surgical outcomes, and minimal trauma, LIHR has become the preferred surgical approach^2,3^.

The introduction of laparoscope-assisted single-needle percutaneous extraperitoneal closure (LSPEC) represents a surgical approach refined from early laparoscopic techniques, as reported by some researchers, which rapidly gained approval due to its efficiency and minimally invasive characteristics^4,5^, this surgical method, however, does not preclude certain inevitable complications, particularly those prone to manifest in the early stages of the learning curve, including recurrence and changes in testicular position^6–9^. A meta-analysis conducted by Isabel et al.^10^, which comprising 27 articles and involving 91,653 patients (26,920 laparoscopic surgery patients and 64,733 open surgery patients), yielded the following results: The average recurrence rate for laparoscopic surgery was 1.57% (range: 0–6%), while for open surgery, it was 1.34% (range: 0–7.8%). The average incidence of cryptorchidism for laparoscopic hernia repair was 0.01% (range: 0–0.1%), whereas for open hernia repair, it was 1.53% (range: 0–6.8%).

Given the substantial incidence of this ailment and the corresponding patient volume, even a low complication rate results in a significant number of children facing these complications. Consequently, there persists an urgent need for research aimed at refining the surgical methodologies for inguinal hernia repair.

Building upon prior investigations into surgical techniques and drawing from a wealth of clinical experience, our study integrates a series of enhancements to LSPEC. We hypothesize that these refinements contribute to an improved therapeutic outcome for this condition without introducing additional complications. This hypothesis is systematically evaluated through a comprehensive review of extensive clinical data. The primary objective of this study is to elucidate these refinements, with the overarching goal of optimizing the treatment of inguinal hernia in pediatric patients and diminishing the incidence of surgical complications.

## Materials and methods

### Data and patients

A retrospective analysis was performed on 809 patients admitted to two prominent pediatric medical centers for inguinal hernia between June 2020 and June 2022. Approval for this study was obtained from the Ethics Committee of Women and Children’s Hospital of Chongqing Medical University, ensuring compliance with the 1964 Helsinki Declaration and its subsequent amendments, along with comparable ethical standards^11^. Written informed consent for the anonymous use of patient data in statistical analysis was obtained from the guardians of the hospitalized children. The analysis covered demographic data, surgical outcomes, and intraoperative conditions.

### Surgical method

Following general anesthesia, an approximately 5 mm incision is made around the umbilicus. Pneumoperitoneum is established using an insufflation needle, which is subsequently removed. A 5 mm trocar is inserted through the incision, allowing for the subsequent introduction of the laparoscope (STORZ^®^ [KARL STORZ Endoscopy (Shanghai, China) Ltd., KARL STORZ China Center, Shanghai, China, 5 mm 30°]). The needle (WHQ-1.6B™, EAGLEFIT^®^, Guangdong, China) was inserted above the body surface projection of the internal ring to create the puncture point. A 3 mm stab incision overlying the internal ring on the affected side was made to facilitate the blunt separation of subcutaneous tissue with forceps, thereby easing the subsequent extraction of the hernia sac.

**Figure 1:**
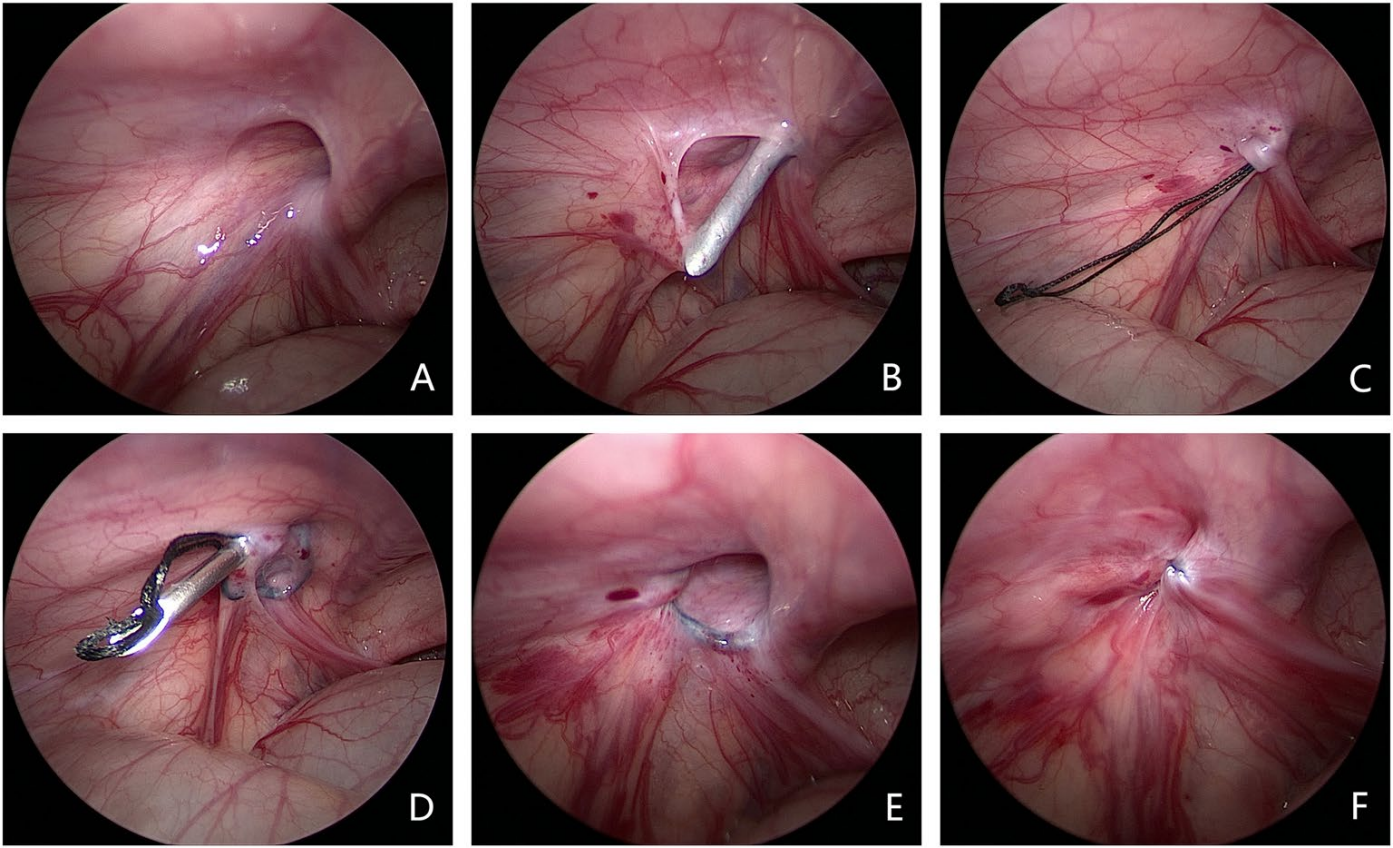
Laparoscopic-assisted hernia sac ligation process.

**Figure 2:**
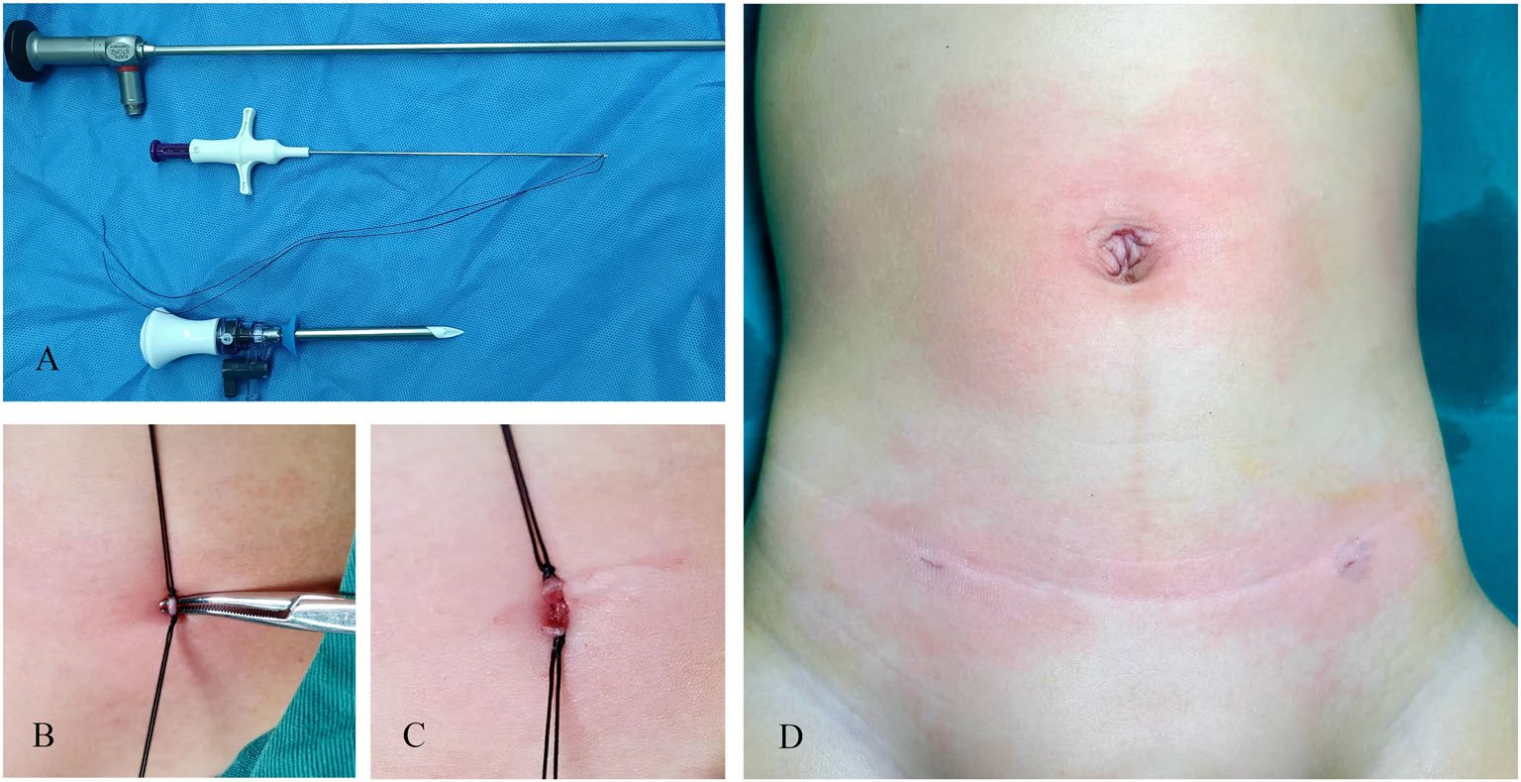
Surgical instruments and extracorporeal hernia sac ligation and dissociation procedure were used (**A**) Surgical instruments; (**B**) Ligate the hernia sac in vitro; (**C**) Detached hernia sac; (**D**) Postoperative appearance).

The suture-carrying needle passed the internal ring via the vas deferens and spermatic vessels, puncturing the peritoneum and leaving the suture. A similar procedure was repeated along the outer side of the internal ring, with the reserved suture being used for complete ligation of the hernia sac (Fig. 1). The two sutures were then divided into upper and lower sections, with each section pulled by a surgeon. The repeated back-and-forth movement separated and elevated the hernia sac, which was subsequently extracted from the body. After ligating the upper and lower ends separately, tissue scissors were used to cut the middle hernia sac, and the two broken sections were then carefully returned to the body. Attention was given to appropriately position the boy's testicles, mitigating the risk of iatrogenic testicular positional changes (Fig. 2).

A subsequent laparoscopic examination confirmed the thorough ligation of the hernia at the internal ring. The ligated internal ring silk line could be visible outside the peritoneum at this point. Confirmation of asymptomatic contralateral internal ring patency prompted prophylactic surgery, which involved repeating the same process^12,13^.

In the video file, we captured the complete and detailed entirety of the surgical procedure (Supplementary Video S1).

### Postoperative management

Postoperatively, discharge timing was determined based on the children's mental response and dietary recovery. A follow-up outpatient review within 1 month after surgery comprised inquiries about clinical symptom relief, routine physical examinations, and ultrasonography to evaluate surgical outcomes.

### Statistical analysis

Descriptive presentation of all patient information included averages and standard deviations. Formal analysis was conducted to further assess the data.

### Ethics approval and consent to participate

Approval of the research protocol by the Ethics Committee of Women and Children’s Hospital of Chongqing Medical University. Written informed consent for participation was signed by the guardian of the child when hospitalized.

## Results

A total of 809 patients, aged 6 to 167 months, with a mean age of 35.0 [19.0–62.5] months, were enrolled in the study. The ligation of 1206 hernia rings (412 unilateral, 397 bilateral) was performed in these patients (Table 1). Clinical diagnosis indicated unilateral hernia in 716 out of 809 patients, with 42% (304/716) of them exhibiting contralateral asymptomatic patency of the processus vaginalis (PPV), resulting in concurrent prophylactic surgery. Consequently, 412 patients underwent unilateral surgery, and 397 underwent bilateral surgery. The mean operation times for the two groups were 15.9 ± 4.8 min and 21.7 ± 3.9 min, respectively. All procedures were conducted laparoscopically, with no instances of conversion to open surgery. Among 12 patients facing procedural challenges solely with puncture needles, 3 mm grasping forceps were used to facilitate the successful completion of the operation. No intraperitoneal organ injuries were observed, except for cases of hemorrhage and hematoma. Incision infection occurred in 7 cases (0.87%). The mean follow-up duration was 20.8 ± 8.6 months. Throughout the follow-up period, no instances of iatrogenic cryptorchidism, testicular atrophy, or recurrence (0%) were detected. Contralateral metachronous inguinal hernia (MCIH) occurred in 2 patients who underwent unilateral surgery (Table 2). All patients in this study were discharged within 1–2 days.

**Table 1:** Demographic and intraoperative clinical characteristics.

Variable	
Number of patients (n)	809
Age: month (IQR)	35.0 [19.0–62.5]
Gender (boy/girl)	628/181
Preoperative laterality (unilateral/bilateral)	716/93
Length of hospital stay, hour, median (IQR)	17.0 [13.0–23.0]
Operation duration, minutes (mean ± SD)	
Unilateral	15.9 ± 4.8
Bilateral	21.7 ± 3.9

*IQR* interquartile range, *SD* standard deviation.

**Table 2:** Comparison of the complications between groups.

Variable	
Follow-up time, months (mean ± SD)	20.8 ± 8.6
Complications, %, n	
Recurrence	0 (0/809)
CMIH (preoperative asymptomatic sides)	0.23 (2/809)
Hematoma	1.61 (13/809)
Ascending testis	0 (0/911)
Testicular atrophy	0 (0/911)
Wound infection	0.87 (7/809)

*CMIH* contralateral metachronous inguinal hernia.

## Discussion

This study presents a novel and modified minimally invasive surgical procedure for pediatric inguinal hernia, which has been proven to be safe and effective in a substantial sample size. Historically, open surgery has been considered the gold standard for pediatric inguinal hernia treatment^14,15^. This method allowed for clear dissection of the inguinal canal, precise hernia sac localization, and high-level ligation, achieving radical treatment of inguinal hernia. However, due to the associated high trauma and the failure to explore the contralateral side^3^, laparoscopic surgery rapidly replaced open surgery. Laparoscopic hernia sac ligation has become the primary surgical approach for pediatric inguinal hernia, with various reported methods^10,16,17^. Laparoscope-assisted Single-needle Percutaneous Extraperitoneal Closure (LSPEC) has gained global acceptance for its rapid and efficient characteristics^4,18^. Nevertheless, this method is not flawless, with reported complications such as a prolonged learning curve and postoperative recurrence^19–21^. Despite their low incidence, considering the high prevalence of the disease, these complications remain a concern for children. Therefore, our center undertook this study to enhance and apply a modified laparoscopic surgery, which amalgamates the advantages of traditional open surgery and laparoscopic surgery, and resulting in satisfactory results in a large sample size. This report aims to contribute to the continual improvement of pediatric inguinal hernia treatment.

In traditional open surgery, complete detachment of the hernia sac is aimed at minimizes the risk of recurrence. However, widely used laparoscopic percutaneous extraperitoneal closure often focuses on high-level ligation outside the peritoneum without hernia sac detachment, potentially increasing the risk of recurrence and hydrocele. Moreover, simple ligation without detachment may lead to changes in testicular or uterine position, which are particularly notable in children with large hernia sacs. Although current studies do not elucidate the long-term reproductive function effects of these changes, they undoubtedly present potential risks^22^. Balancing the advantages of open and laparoscopic surgery to reduce the likelihood of various complications remains challenging and valuable.

Our surgical improvement, rooted in laparoscopic surgery, alters the hernia sac ligation method by combining ligation and detachment under minimally invasive conditions. This approach minimizes the recurrence risk and avoids potential risks to the testicle or uterus position. The core step involves completely encircling the hernia sac with a crochet hook and freeing it with a double femoral line pullback. This method ensures maximal ligation of the hernia sac at its highest position, reducing the risk of complications. These minor improvements do not increase surgical difficulty, thus significantly decreasing the learning curve and postoperative complication incidence for surgeons. Compared to traditional surgery, our approach is minimally invasive, thereby overcoming the unclear anatomy limitations of open surgery. In contrast to common LSPEC, our method maximally reduces recurrence and complications(such as recurrences caused by suture slippage and suture reactions resulting from superficial ligations^23^) related to organ position changes, thus representing a safe and valuable improvement.

The main concerns regarding the application of our technique involve the possibility of testicular blood flow obstruction due to division of the PPV and the potential elevation of testicular position caused by adhesions. Our surgical technique represents a modified version of laparoscopic surgery. A randomized controlled trial reported a significant decrease in testicular perfusion and size in 3.3% of patients in the open group, while there was no difference in pre- and post-operative ultrasound findings in the laparoscopic group^24^. This difference may be attributed to the magnification advantage provided by laparoscopy, enabling more precise surgical manipulation and thus greater preservation of testicular vessels. Additionally, the encircling of the hernia sac is performed under laparoscopic guidance, and subsequent traction of the sac for extracorporeal ligation and division does not result in additional vascular disruption. On the contrary, after extracorporeal traction, we are able to observe whether there are any vascular attachments at the ligated portion before further ligation and division, further reducing the risk of vascular injury. Furthermore, downward traction of the affected testicle post-ligation helps to prevent changes in testicular position. Reviewing the clinical data of children undergoing this modified surgery and summarizing follow-up results, our study reveals no increase in surgical duration, as well as no occurrences of iatrogenic cryptorchidism, testicular atrophy, or recurrence during the follow-up period, distinguishing it from other studies^3–5,25,26^.

In addition, based on our clinical experience, we advocate the use of a 3 mm auxiliary forceps in specific pediatric groups, especially. The multiple folds in the hernia sac make it challenging to pass the cannula, and simple ligation may not completely prevent hernia recurrence or hydrocele formation. Moreover, for newborn female hernias, the shorter length of the round ligament brings the ovary and fallopian tube closer to the internal inguinal ring^27^. Hence, the use of assisted forceps is necessary to pull the round ligament to complete the ligation of the hernia sac. This adds to the complexity and risks of surgery. The auxiliary forceps aid in quick, safe, and complete hernia sac ligation, thereby reducing surgical difficulty and anesthesia time. We recommend routine preparation of 3 mm auxiliary forceps and their utilization based on hernia sac exploration. Additionally, auxiliary forceps are beneficial for contralateral exploration, aiding in reducing the occurrence of contralateral metachronous inguinal hernia (CMIH)^3,5^.

Admittedly, the study has limitations, including its retrospective nature and the fact that data was sourced from only two centers. Future prospective studies will be conducted to validate the effectiveness of this procedure.

## Conclusions

This study retrospectively reviewed clinical data from 809 cases of pediatric patients undergoing this modified surgical procedure, which demonstrated its superior efficacy in reducing postoperative recurrence. Furthermore, compared to other procedures, the modified surgical procedure did not result in an increased incidence of additional complications. Therefore, the efficacy of this modified surgical procedure is commendable, and we recommend that fellow practitioners adopt it to pursue better surgical outcomes.

### Supplementary Information

Supplementary Information.

Supplementary Video 1.

## Data Availability

The datasets used and/or analysed during the current study available from the corresponding author on reasonable request.
