# Supplementary material for: Outcomes from a new modified single needle laparoscopic percutaneous extraperitoneal closure and cut off for pediatric inguinal hernia
Source: Sci Rep. 2024 May 26;14:12029. doi: 10.1038/s41598-024-62769-7 (PMC11128452; doi:10.1038/s41598-024-62769-7)
Supplement: Supplementary file 1 — Supplementary Information. [file 41598_2024_62769_MOESM1_ESM.pdf]

STROBE Statement—checklist of items that should be included in reports of observational studies

	Item No.	Recommendation	Page No.	Relevant text from manuscript
**Title and abstract**	1	(*a*) Indicate the study's design with a commonly used term in the title or the abstract	1	Line1-3
		(*b*) Provide in the abstract an informative and balanced summary of what was done and what was found	3	Line81-94
**Introduction**				
Background/rationale	2	Explain the scientific background and rationale for the investigation being reported	4	Line104-109
Objectives	3	State specific objectives, including any prespecified hypotheses	4	Line110-115
**Methods**				
Study design	4	Present key elements of study design early in the paper	5	Line128-137
Setting	5	Describe the setting, locations, and relevant dates, including periods of recruitment, exposure, follow-up, and data collection	5	Line128-137
Participants	6	(*a*) *Cohort study*—Give the eligibility criteria, and the sources and methods of selection of participants. Describe methods of follow-up<br>*Case-control study*—Give the eligibility criteria, and the sources and methods of case ascertainment and control selection. Give the rationale for the choice of cases and controls<br>*Cross-sectional study*—Give the eligibility criteria, and the sources and methods of selection of participants	5	Line128-137
		(*b*) *Cohort study*—For matched studies, give matching criteria and number of exposed and unexposed<br>*Case-control study*—For matched studies, give matching criteria and the number of controls per case	None	
Variables	7	Clearly define all outcomes, exposures, predictors, potential confounders, and effect modifiers. Give diagnostic criteria, if applicable	5	Line128-137
Data sources/ measurement	8*	For each variable of interest, give sources of data and details of methods of assessment (measurement). Describe comparability of assessment methods if there is more than one group	5	Line128-137
Bias	9	Describe any efforts to address potential sources of bias	None	
Study size	10	Explain how the study size was arrived at	None	

Continued on next page

Quantitative variables	11	Explain how quantitative variables were handled in the analyses. If applicable, describe which groupings were chosen and why	None	
Statistical methods	12	(*a*) Describe all statistical methods, including those used to control for confounding	6-7	Line167-169
		(*b*) Describe any methods used to examine subgroups and interactions	6-7	Line167-169
		(*c*) Explain how missing data were addressed	None	
		(*d*) *Cohort study*—If applicable, explain how loss to follow-up was addressed<br>*Case-control study*—If applicable, explain how matching of cases and controls was addressed<br>*Cross-sectional study*—If applicable, describe analytical methods taking account of sampling strategy	6	Line117-118
		(*e*) Describe any sensitivity analyses	None	
**Results**				
Participants	13*	(a) Report numbers of individuals at each stage of study—eg numbers potentially eligible, examined for eligibility, confirmed eligible, included in the study, completing follow-up, and analysed	7	Line172-186
		(b) Give reasons for non-participation at each stage	7	Line172-186
		(c) Consider use of a flow diagram	None	
Descriptive data	14*	(a) Give characteristics of study participants (eg demographic, clinical, social) and information on exposures and potential confounders		Table 1
		(b) Indicate number of participants with missing data for each variable of interest		Table1
		(c) *Cohort study*—Summarise follow-up time (eg, average and total amount)		Table2
Outcome data	15*	*Cohort study*—Report numbers of outcome events or summary measures over time	None	
		Case-control study—Report numbers in each exposure category, or summary measures of exposure	None	
		Cross-sectional study—Report numbers of outcome events or summary measures	None	
Main results	16	(*a*) Give unadjusted estimates and, if applicable, confounder-adjusted estimates and their precision (eg, 95% confidence interval). Make clear which confounders were adjusted for and why they were included	None	
		(*b*) Report category boundaries when continuous variables were categorized	None	
		(*c*) If relevant, consider translating estimates of relative risk into absolute risk for a meaningful time period	None	

Continued on next page

Other analyses	17	Report other analyses done—eg analyses of subgroups and interactions, and sensitivity analyses	None	
**Discussion**				
Key results	18	Summarise key results with reference to study objectives	10	Line235-239
Limitations	19	Discuss limitations of the study, taking into account sources of potential bias or imprecision. Discuss both direction and magnitude of any potential bias	10	Line251-253
Interpretation	20	Give a cautious overall interpretation of results considering objectives, limitations, multiplicity of analyses, results from similar studies, and other relevant evidence	10	Line235-239
Generalisability	21	Discuss the generalisability (external validity) of the study results	10	Line240-251
**Other information**				
Funding	22	Give the source of funding and the role of the funders for the present study and, if applicable, for the original study on which the present article is based	None	

*Give information separately for cases and controls in case-control studies and, if applicable, for exposed and unexposed groups in cohort and cross-sectional studies.

Note: An Explanation and Elaboration article discusses each checklist item and gives methodological background and published examples of transparent reporting. The STROBE checklist is best used in conjunction with this article (freely available on the Web sites of PLoS Medicine at http://www.plosmedicine.org/, Annals of Internal Medicine at http://www.annals.org/, and Epidemiology at http://www.epidem.com/). Information on the STROBE Initiative is available at www.strobe-statement.org.
